# A qualitative exploration of women’s experiences, stigma, and access to care in gaming disorder

**DOI:** 10.1192/j.eurpsy.2026.12231

**Published:** 2026-06-15

**Authors:** Catia Martins Castro, Noelle Marino Rothlisberger, Daniele Zullino, Sophia Achab

**Affiliations:** 1 WHO Collaborating Centre for Training and Research in Mental Health, Geneva, Switzerland; 2 Department of Psychiatry, Faculty of Medicine, University of Geneva, Switzerland

**Keywords:** access to care, behavioral addiction, gaming disorder, stigma, women, women’s health

## Abstract

**Background:**

Gaming disorder (GD) is recognized by the WHO as a behavioral addiction, yet research remains predominantly men-centric. This study explores the experiences of women gamers, their perceptions of GD, and the barriers women face in accessing care.

**Methods:**

Semi-structured interviews were conducted with seven participants identifying as women (six cisgender women and one transgender woman) aged 21–26 years old, in France and Switzerland. Inductive reflexive thematic analysis was used to identify women’s experiences of gaming behavior, stigma, and help-seeking.

**Results:**

Participants reported diverse gaming behaviors, with preferences for narrative-driven games (e.g., RPG and simulations). Stigma, lack of self-awareness, limited self-recognition of problematic gaming, and broader societal misconceptions emerged as significant barriers to recognizing GD as a potential clinical issue warranting help-seeking. None had sought professional care for GD, citing gendered stereotypes and diagnostic biases as key deterrents.

**Conclusions:**

The study highlights the need for women-centered diagnostic tools, clinical approaches, targeted awareness campaigns, and flexible support systems for women with GD. Future research should prioritize larger, diverse samples and intersectional approaches.

## Introduction

In recent years, digital technologies have become deeply embedded in everyday life, with video games emerging as one of the most widespread forms of entertainment, further expanding through increasing accessibility and technological innovations such as mobile gaming, virtual reality, and cross-platform play. However, alongside these developments, concerns have emerged about the potential negative consequences of use.

Characterized by impaired control over gaming, prioritization of gaming over other activities, and continuation despite negative consequences, gaming disorder (GD) is recognized by the World Health Organization (WHO) in the International Classification of Diseases *11th* Revision (ICD-11) as a behavioral addiction [1]. While global prevalence estimates range from 1 to 3%, research on GD has overwhelmingly focused on young male populations, potentially limiting understanding of how GD manifests and its impact on women [2–4]. This imbalance reflects broader cultural stereotypes that frame gaming as a predominantly male activity, influencing GD research, as well as diagnostic biases in tools like the Internet Gaming Disorder Scale–Short-Form (IGDS9-SF) [5], which may not adequately capture women’s symptoms [6–9].

Gender dynamics within gaming environments have been widely documented in the literature. Women players frequently encounter persistent gender stereotypes, including the underrepresentation and sexualization of women characters, as well as narratives that portray women in secondary or victimized roles (6,10,11). These representations contribute to broader social dynamics in gaming communities, where women’s skills may be questioned and where they may experience sexist or stereotypical comments during gameplay (10,11,12). Such gendered dynamics can shape gaming experiences and preferences. Empirical studies suggest that women tend to engage more frequently with genres such as puzzle, simulation, adventure, or casual games, often playing for entertainment or leisure, and typically spending less time gaming than men (6,11). Men, by contrast, more often report competitive motivations, achievement-oriented play, and greater time investment, frequently within genres such as MMORPGs, MOBAs, or first-person shooters (6,11). However, these differences are influenced by broader sociocultural factors, including gendered marketing and gaming environments, and should not be interpreted as fixed behavioral patterns (6,10).

Women in gaming are confronted with double sexism: they are stigmatized as gamers in a male-dominated culture and often excluded from mainstream discussions on GD [10–12]. Consequently, women’s experiences of gaming and gaming-related difficulties have received comparatively less research attention, contributing to an important gap in understanding how women experience, perceive, and navigate GD, as well as the barriers they face in accessing care [12, 13].

In the present study, gender is understood as a sociocultural construct that shapes social roles, identity, norms, exposure to stigma, and help-seeking trajectories. In the context of GD, gender is clinically relevant because it may influence symptom expression, self-recognition of impairment, interactions with gaming communities, and access to diagnosis and treatment. A gender-sensitive perspective regarding women’s care is therefore essential not only for descriptive accuracy, but also for equitable clinical decision-making. Specifically, approaches considering the gender specificity are increasingly recognized as essential for understanding behavioral addictions, particularly in domains historically dominated by male samples, such as gaming research [6, 9, 11].

Existing studies have predominantly relied on quantitative designs and male-dominated samples, often overlooking the gendered dimensions of gaming experiences. While previous studies have examined women’s participation in gaming cultures and gender differences in gaming behavior, relatively scarce qualitative research has explored how women themselves interpret potentially problematic gaming and the barriers they encounter when recognizing GD or seeking support [6]. Addressing this gap by providing an in-depth, experiential exploration of women’s perspectives on GD is important for developing more effective, targeted clinical frameworks and public health responses.

This study aims to provide an in-depth exploration of women gamers’ experiences, gaming motivations, stigma within gaming cultures, and the barriers they face in recognizing symptoms of GD and accessing care. To this end, the study seeks to:

1. Explore women gamers’ experiences of GD, including their motivations, behaviors, and self-perception within the gaming community.
2. Identify women’s barriers to recognizing GD and accessing care, such as stigma, diagnostic biases, and lack of awareness.
3. Discuss implications for clinical practice, public health, and gaming culture, advocating for a more inclusive and equitable approach to GD research and treatment.

Because the present study focuses on women participants, the aim is not to compare gender differences but rather to explore women’s gendered experiences of gaming, including stigma, identity negotiation, and barriers to help-seeking within gaming cultures.

## Methods

### Study design

This study employed a qualitative research design to explore the experiences, perceptions, and barriers related to GD among women gamers.

#### Ethics

Ethical approval and oversight followed the institutional guidelines of the University of Geneva. All participants received detailed information about the study and provided informed consent before participation. Participation was voluntary, and participants could withdraw at any time. All data were anonymized to ensure confidentiality. Participants were assured that their responses would remain anonymous, and that pseudonyms or numerical identifiers would be used in transcripts and publications. Interviews were audio-recorded with permission and securely stored in accordance with institutional data protection standards. The study adhered to the Consolidated Criteria for Reporting Qualitative Research (COREQ) [14] guidelines to ensure transparency and methodological rigor and complied with the ethical principles outlined in the Declaration of Helsinki and the conventions governing research involving human participants.

### Participants

#### Recruitment and sampling

Participants were recruited through purposive sampling using an open and voluntary recruitment strategy based on word-of-mouth and professional networks, targeting individuals who self-identified as women and had experience with video games. Although the recruitment channels had the potential to reach a broader population, the final sample consisted of seven participants who expressed interest and met the inclusion criteria during the data collection period.

Eligibility criteria required participants to be adult women who regularly played video games (more than 10 hours per week during the past year) with diverse gaming preferences in order to maximize sample heterogeneity. Seven participants, identifying as women, aged between 21 and 26 years old, and residing in France or Switzerland, were recruited. The sample included six cisgender women and one transgender woman, reflecting the diversity of gendered experiences within contemporary gaming communities. Their demographics are summarized in Table 1.

**Table 1.** Demographic characteristics of the women gamer participants Table 1. long description.

Participant	Age	Occupation	Preferred games	Platform	Weekly gaming hours	Self-identifies as “gamer”?
1G	23	Student (Medicine)	RPG, Visual Novels	Console	12–15	No
2G	25	Student (Game Art)	Simulation, Strategy	PC	15–20	Yes
3G	24	Student	MMORPG, FPS	Console	10–14	Partially
4G	26	Psychologist	RPG, Otome Games	PC	18–22	Yes
5G	22	Student	FPS, MOBA	PC	20–25	Yes
6G	21	Student	Simulation, Puzzle	Console	8–12	No
7G	23	Student	RPG, Adventure	PC	14–18	Partially

Given the exploratory nature of the study and the focus on in-depth qualitative understanding, a small purposive sample was considered appropriate. Participation was voluntary, and no financial compensation was provided.

In accordance with the Sex and Gender Equity in Research (SAGER) guidelines, the term sex refers to biological attributes associated with female and male bodies, whereas gender refers to socially constructed roles, behaviors, and identities [15]. In the present study, participants were recruited based on gender.

The sample size is consistent with qualitative research aiming for depth rather than breadth, allowing for rich, idiographic exploration of participants’ lived experiences, with designs that prioritize depth of exploration rather than statistical representativeness. Qualitative studies frequently rely on smaller samples in order to generate rich, contextualized accounts of participants’ experiences [16]. Moreover, recent methodological frameworks suggest that the adequacy of qualitative samples should be assessed in terms of *information power*, whereby fewer participants may be sufficient when the study aim is focused, the participants are relevant to the research question, and the data generated are rich and detailed [17].

### Data collection

Semi-structured interviews were conducted, three face-to-face and four via videoconferencing (Zoom), depending on participants’ availability and location. Interviews lasted ~60–90 minutes. The same predefined semi-structured interview guide was used across all interviews. No noticeable differences were observed in the depth, richness, or quality of the data obtained between the online and face-to-face formats. All interviews were audio-recorded with participants’ consent and subsequently transcribed verbatim. All interviews were transcribed using an AI-based transcription software compliant with General Data Protection Regulation (GDPR) standards. Audio files were pseudonymized before upload, and no identifiable information was processed or retained by the provider [18].

### Data analysis

Data were analyzed inductively using reflexive thematic analysis [19] to identify patterns in the data. This qualitative approach is designed to identify and interpret patterns of meaning across participants’ accounts, focusing on their lived experiences and the meanings they attribute to them. The analysis followed Braun and Clarke’s six-phase framework [16, 19]. First, transcripts were read and re-read to ensure familiarity with the data and to identify initial points of interest. Second, line-by-line coding was conducted, capturing both semantic (explicit) and latent (interpretative) meanings. Initial coding was led by the second author, with themes subsequently reviewed and refined through reflexive discussions with the wider research team. Third, codes were iteratively organized into preliminary themes based on conceptual similarities and relevance to the research questions. In the fourth and fifth phases, themes were reviewed, refined, and clearly defined through ongoing discussions within the research team, ensuring coherence and consistency across the dataset. Rather than relying on inter-rater reliability, analytic rigor was achieved through collaborative, reflexive discussions, in which interpretations were critically examined and refined. In the final phase, themes were fully developed and illustrated using representative excerpts from participants’ narratives. Throughout the process, reflexivity was maintained, with the researchers critically considering how their perspectives and assumptions may have shaped the analytical process.

**Figure 1.**
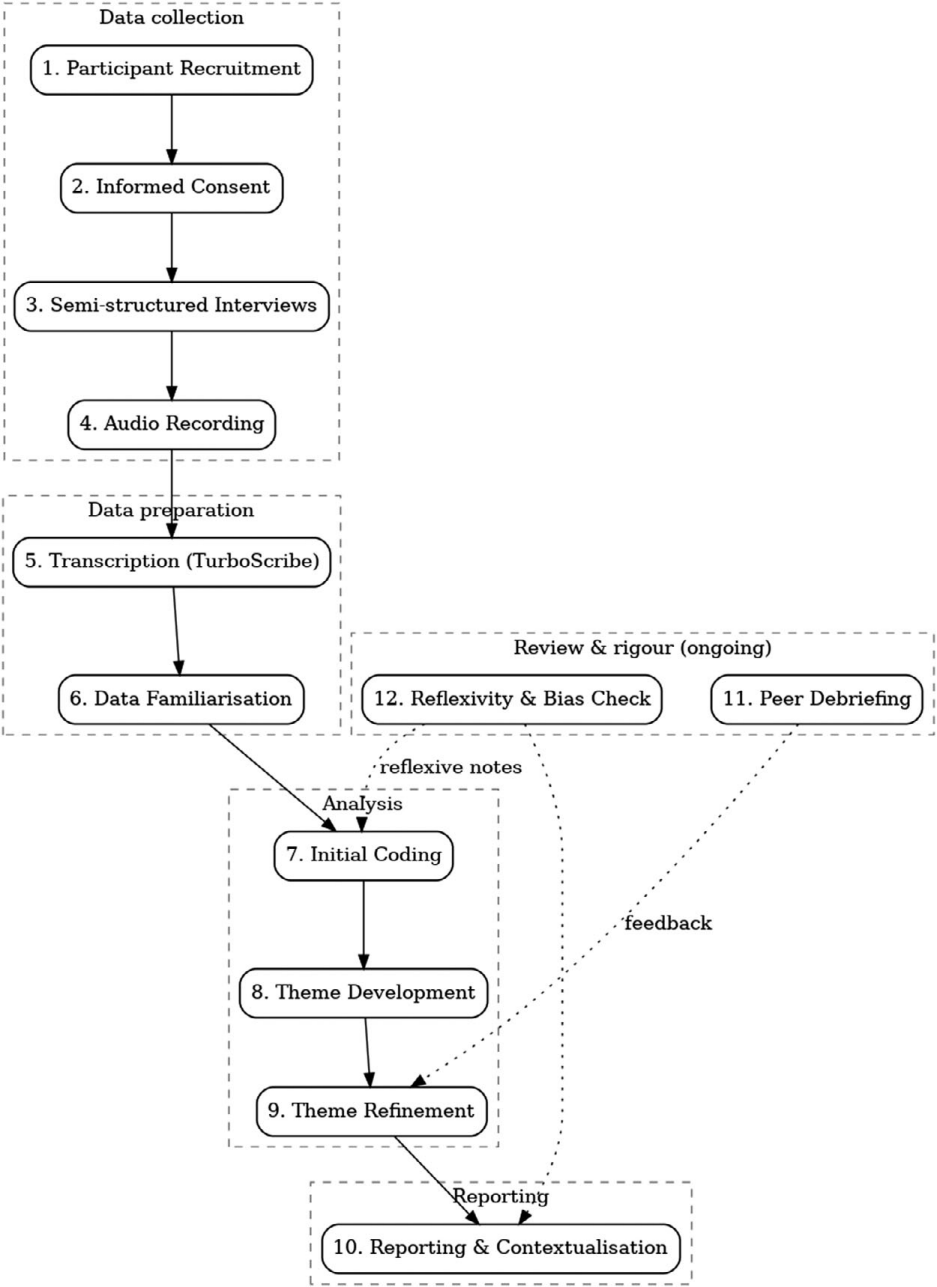
Reflexive thematic analysis workflow illustrating data collection, preparation, analysis, reporting, and ongoing processes to ensure rigor. *Note:* Transcription was performed with TurboScribe (2024)[18]. Peer debriefing and reflexivity occurred throughout; dotted arrows indicate iterative feedback loops. Figure 1. long description.

A schematic overview of the analytic process is illustrated in Figure 1.

## Results

The inductive reflexive thematic analysis of interviews with seven women gamers (aged 21–26 years) revealed three overarching themes that capture their experiences of gaming, perceptions of GD, and barriers to recognizing and addressing problematic gaming behaviors. These themes are: (1) Identity and stigma in gaming culture, (2) Gaming behaviors and motivations, and (3) Barriers to recognizing GD and accessing care. Each theme is presented below, supported by illustrative excerpts from participants’ narratives and situated within the broader literature on GD and gender.

Indicative prevalence is reported to provide a sense of how widely themes were represented across participants; however, consistent with reflexive thematic analysis, these descriptive and do not imply statistical generalization. An overview of themes and subthemes is presented in Table 2.

**Table 2.** Themes, subthemes, and dataset support across participants Table 2. long description.

Theme	Subtheme	Dataset support across participants
Identity and stigma in gaming culture	Self-identification as a “gamer”	Yes = 3; Partially = 2; No = 2
	Sexism and exclusion in gaming spaces	Reported across several interviews (5)
Gaming behaviors and motivations	Preference for at least one narrative-driven or less competitive genre (e.g., RPG, visual novel, simulation, puzzle, adventure, otome)	5/7
	Gaming used for emotional coping, escape, or stress reduction	Reported across several interviews
Barriers to recognizing GD and accessing care	Prior familiarity with the term GD before the interview	None reported (0/7)
	Previous professional help-seeking for GD	None reported (0/7)
	Perceived lack of tailored resources for women with GD	Reported across several interviews

*Note*: Exact counts are reported only where directly supported by participant characteristics or explicit statements in the dataset. For other subthemes, prevalence is described qualitatively to remain consistent with reflexive thematic analysis.

### Identity and stigma in gaming culture

Most participants described experiences of stigma in gaming environments, particularly in relation to women’s gendered expectations and negative interactions. The notion of what it means to identify as a “gamer” emerged as a complex and often contested issue. Only three participants explicitly identified as “gamers,” while the others expressed ambivalence or rejection of the label. This reluctance was closely linked to gendered stereotypes positioning gaming as a predominantly male activity. For example, Participant 6G (age 21, student) stated, “*I don’t call myself a gamer because I feel like that term is for people who play competitively online, and that’s not me.*” This sentiment reflects the broader cultural perception that “real gamers” are those who engage in competitive, time-intensive genres, such as Massively Multiplayer Online Role-Playing Games (MMORPGs) or First-Person Shooter (FPS) games, spaces traditionally dominated by men [10, 20].

Several participants (*n* = 5) reported encountering sexist attitudes, including dismissive comments and exclusion from gaming communities. Participants also described experiencing double sexism, both as women in gaming communities and gamers in a broader society, within a male-dominated space that frequently stigmatizes gaming. Several women reported being labeled as “*noobs*” (e.g., novice, inexperienced gamers) or “*pick-me girls*” (e.g., by male peers, terms that undermine their legitimacy and reinforce the idea that women are either inexperienced or seeking male attention). Participant 3G (age 24, Student) shared: “*When I play online and guys hear my voice, they assume I’m bad at the game. They’ll say things like, ‘Oh, you’re a girl? Let me carry you.’ It’s frustrating because I’ve been gaming for years, but I’m automatically seen as less skilled.*”

This aligns with existing research highlighting the hyper sexualization and marginalization of women in gaming communities [6]. The stigma extended beyond online interactions. Some participants described negative perceptions from family and friends. Participant 1G (age 23, Student in Medicine) noted: “*My parents still think gaming is a waste of time. They don’t understand that it’s a hobby like any other, but because I’m a woman, they assume I should be doing something more ‘productive’ or ‘feminine.’*”

These experiences contributed to a sense of exclusion from both gaming communities and mainstream discussions about GD, creating additional barriers for women who might otherwise seek help for problematic gaming behaviors.

### Gaming behaviors and motivations

Participants reported diverse gaming behaviors, with a marked preference for narrative-driven, single-player games such as RPGs, visual novels, and simulations. Unlike the competitive, multiplayer genres often associated with GD in men, these games were described as less stressful and more immersive. Participant 4G (age 26, Psychologist) explained: “*I prefer RPGs because I can play at my own pace and focus on the story. I don’t like the toxicity of online multiplayer games, so I avoid them.*”

This preference for less competitive, more story-focused games may explain why women are underrepresented in GD research, which has historically focused on genres like MMORPGs and FPS games [6, 20–22]. However, despite their choice of game genres, some participants still reported periods of excessive gaming, particularly during times of stress or emotional distress. Participant 7G (age 23, Student) described gaming as a coping mechanism:

“*When I’m anxious or overwhelmed, I’ll play for hours just to escape. It’s not about the game itself, it’s about not having to think about everything else.*”

Motivations for gaming varied but often included escape, social connection, and skill development. For instance, Participant 2G (age 25, Student in Game Art) used gaming to bond with friends and explore creative interests: “*I love games like Animal Crossing* because I can design my own world. It’s relaxing, and I can play with my friends without dealing with the toxicity you find in other games.”

Notably, none of the participants had sought professional help for GD, even when they acknowledged that their gaming habits sometimes interfered with daily responsibilities. Instead, they relied on self-regulation strategies, such as setting personal limits or playing with friends to stay accountable.

### Barriers to recognizing GD and accessing care

A lack of awareness about GD emerged as a central barrier. None of the participants were familiar with the term GD before the interview and assumed their gaming behavior was normal or harmless. Furthermore, the lack of self-recognition of one’s gaming as potentially problematic, and recognition within diagnostic and care contexts, including possible under-identification by professionals when women’s experiences do not match dominant stereotypes of GD, was a significant barrier to recognizing problematic gaming behaviors. None of the participants were familiar with the term “GD” before the interview, and most assumed that their gaming habits were normal or harmless. Participant 5G (age 22, Student) admitted: “*I didn’t realize gaming could be an addiction. I thought it was just something people did for fun, not something that could be a problem.*”

This lack of awareness was compounded by diagnostic biases in existing GD tools, which participants felt did not reflect their experiences. For example, the IGDS9-SF, a commonly used screening tool, emphasizes competitive gaming and social conflict symptoms more commonly reported by men [11, 23]. Participant 4G (Psychologist) critiqued this bias: “*Most of the questions on those scales are about things like ‘neglecting responsibilities’ or ‘conflicts with others.’ But for me, gaming is more about emotional regulation. I don’t fit the stereotype of a ‘gaming addict,’ so I never thought I might have an issue.*”

Stigma also played a critical role in preventing participants from seeking help. Fear of being judged, both as gamers and as women with a potential behavioral addiction, led to self-silencing and avoidance of professional support. Participant 3G explained: “*I’d be embarrassed to talk to a therapist about gaming. They’d probably think I’m just lazy or immature.*”

Finally, participants highlighted the lack of resources for women with GD. Existing support systems, such as online forums or treatment programs, were perceived as male-centric and unwelcoming. Participant 6G suggested: “*If there were groups for women, or even just more information about how GD affects women differently, I think more of us would reach out for help.*”

## Discussion

While the study sample consists of women participants, the experiences described by them are influenced by a broader gendered norm within gaming culture, including stereotypes about who is considered a legitimate gamer and how gaming behaviors are socially interpreted.

The findings provide important insights into women gamers’ experiences of GD and the gendered barriers that contribute to its under-recognition and undertreatment of GD in women.

By centering the voices of seven women gamers, this study challenges the male-dominated narrative of GD and underscores the need for women-sensitive approaches in diagnosis, treatment, and public health strategies. Below, we discuss the implications of our findings in the context of existing literature, clinical practice, and future research.

### Gender disparities in GD research and diagnosis

The results of this study align with previous research, indicating that women are underrepresented in GD studies [3, 6]. The preference for narrative-driven, single-player games among our participants contrasts with the competitive, multiplayer genres typically associated with GD in men [4, 24]. These findings are consistent with quantitative evidence showing that women are more frequently represented in profiles where gaming serves as a leisure or emotion-regulation strategy rather than a highly social or competitive activity [25, 26].

Our findings suggest that current diagnostic tools, such as the IGDS9-SF, may not adequately capture the diverse manifestations of GD in women. For example, the IGDS9-SF emphasizes symptoms like neglecting responsibilities and social conflict, which are less commonly reported by women [27]. Instead, women in our study described gaming as a coping mechanism for stress and emotional distress, a motivation that is not fully reflected in existing screening tools. This diagnostic bias has significant implications for prevalence estimates and clinical practice. If GD is primarily framed through a male-centric lens, women may be less likely to recognize their own problematic gaming behaviors or seek help. As Participant 4G (Psychologist) noted, the lack of alignment between women’s experiences and diagnostic criteria can lead to self-silencing and avoidance of treatment. To address this, future research should prioritize the development of women-inclusive diagnostic tools that account for the emotional and social functions of gaming in women.

### Stigma and double sexism in gaming culture

The stigma and double sexism faced by women gamers emerged as a central theme in our study. Participants described being labeled as “*noobs*” or “*pick-me girls*,” terms that reflect the hyper sexualization and marginalization of women in gaming communities [28]. This stigma extends beyond online interactions, with some women reporting judgment from friends and family who perceive gaming as a “waste of time” or “unfeminine” hobby. Such attitudes contribute to a sense of exclusion from both gaming communities and mainstream discussions about GD, further isolating women who might benefit from support.

The cultural perception of gaming as a male activity also influences how women engage with gaming and perceive their own behaviors. For instance, Participant 6G’s reluctance to identify as a “gamer” reflects the broader societal assumption that “real gamers” are competitive, time-intensive players, traits traditionally associated with masculinity. This gendered stereotype not only undermines women’s legitimacy as gamers but also discourages them from seeking help for problematic gaming behaviors. Clinicians and researchers must recognize that stigma is a significant barrier to care and work to create inclusive, nonjudgmental spaces where women feel comfortable discussing their gaming habits.

### Emotional regulation and gaming as a coping mechanism

A notable finding of this study is the role of gaming as a coping mechanism for stress and emotional distress. Several participants described using gaming to escape negative emotions or manage anxiety, particularly during periods of high stress. This aligns with research suggesting that emotional regulation is a key motivation for gaming in women [20, 25–27] However, unlike men, who are more likely to use gaming for competition and achievement, women in our study emphasized the immersive and relaxing aspects of narrative-driven games.

This distinction has important implications for treatment and prevention strategies. This preference for narrative-driven and less competitive games is consistent with recent person-centered research examining psychological profiles of gamers. Four latent profiles were examined based on emotional regulation and motivational factors across all game types [25, 26]. This large-scale quantitative study [25] suggests that profiles with higher proportions of women displayed motivational patterns centered on escapism, fantasy, and recreation, reinforcing our qualitative finding that women players often view gaming as a leisure activity or coping strategy, rather than a competitive pursuit. The use of gaming as an emotional buffer and the existing GD interventions that often focus on reducing gaming time or addressing social conflicts, our findings suggest that emotional regulation should be a central component of GD treatment for women. Clinicians should explore alternative coping strategies (e.g., mindfulness and creative outlets) and address the underlying emotional needs that gaming fulfills. For example, Participant 7G’s use of gaming to “not think about everything else” highlights the need for trauma-informed and emotion-focused therapies in GD treatment.

### Barriers to recognizing GD and accessing care

The lack of awareness about GD was a significant barrier to recognizing problematic gaming behaviors. None of the participants was familiar with the term GD before the interview, and most assumed that their gaming habits were normal or harmless. This lack of awareness was compounded by the absence of women-centered resources, such as support groups or educational materials tailored to women.

The stigma associated with GD also played a critical role in preventing participants from seeking help. Fear of being judged, both as gamers and as women with a potential behavioral addiction, led to self-silencing and avoidance of professional support. Participant 3G’s concern that a therapist would view her as “*lazy or immature*” reflects the broader societal stigma surrounding behavioral addictions, particularly in women. To overcome this barrier, public health campaigns should aim to normalize discussions about GD and reduce shame associated with seeking help.

Additionally, participants highlighted the lack of women-sensitive resources for women with GD. Existing support systems, such as online forums or treatment programs, were perceived as men-centric and unwelcoming. To address this, clinicians and policymakers should prioritize the development of women-focused support groups and gender-inclusive treatment programs. Participant 6G’s suggestion of “groups for women” underscores the need for safe, supportive spaces where women can share their experiences without fear of judgment.

### Clinical and public health implications

The findings of this study have several clinical and public health implications:

1. Diagnostic tools: There is an urgent need to revise existing GD screening tools to more effectively capture women’s experiences. This may involve incorporating questions about emotional regulation, escape, and social connection, which are less emphasized in current tools.
2. Clinical practice: Clinicians should be trained to recognize the women’s symptoms of GD and adopt a nonjudgmental, trauma-informed approach to treatment. This includes exploring the emotional and social functions of gaming and addressing the underlying needs that gaming fulfills.
3. Public health campaigns: Efforts to raise awareness about GD should target women specifically, emphasizing that problematic gaming is not limited to competitive or multiplayer genres. Campaigns should also challenge gendered stereotypes about gaming and promote inclusive discussions about GD.
4. Support systems: The development of women-focused support groups and gender-inclusive treatment programs could help reduce the stigma associated with GD and encourage more women to seek help.

Across themes, patterns were consistently observed across the majority of participants, with some variability reflecting individual differences in experiences and interpretations. The results of this study underscore the unique challenges faced by women gamers in recognizing and addressing GD. Women’s gaming behaviors and motivations often differ from those of men, yet diagnostic tools, research, and support systems remain focused on male experiences. The stigma, lack of awareness, and diagnostic biases identified in this study contribute to the under-recognition and undertreatment of GD in women, highlighting the need for women-centered approaches in both research and clinical practice.

Beyond the development of women’s diagnostic tools and support systems, future models of care should adopt a person-centered and women-centered approach to behavioral addictions [29]. Such approaches acknowledge the diversity of motivations and psychosocial contexts shaping women’s experiences of GD and related problematic internet use. An example of women’s person-centered practice can be found in the Swiss ReConnecte Programme, which provides personalized assessment and care for problematic internet use in women [30]. Integrating such perspectives into prevention and treatment frameworks may improve engagement, reduce stigma, and foster more equitable access to care.

This study contributes to the literature by integrating a women-sensitive and an experiential perspective into the understanding of GD, highlighting how emotional regulation, identity, and social context intersect in influencing women’s gaming behavior.

### Limitations and future research directions

To ensure the trustworthiness of the study, several strategies were employed, including triangulation, peer debriefing, and researcher reflexivity throughout the analytic process. These measures enhanced credibility and reduced the risk of individual bias. Nonetheless, the study has limitations that should be acknowledged. The small sample size and relative homogeneity of participants limit the generalizability of the findings, while the reliance on purposive recruitment may have introduced selection bias. Regarding the open recruitment strategy, as the invitation was disseminated through open channels, it was not possible to determine how many individuals were reached or the reasons for nonparticipation. However, qualitative research prioritizes depth of insight rather than representativeness [16], and smaller samples may be sufficient when interviews provide rich and relevant data, in line with the principle of *information power* [17]. Future studies with larger and more diverse samples could further explore women’s experiences of gaming across different contexts.

Additionally, the self-reported nature of the data may introduce biases, such as social desirability or recall errors. Future studies could incorporate mixed-methods approaches, combining qualitative interviews with quantitative measures of gaming behavior and emotional regulation. Because the study included only women participants, the findings cannot be generalized to male or gender-diverse populations [15].

Finally, this study focused on young adult women in France and Switzerland; the small sample size and homogeneity of participants (young, educated women) limit the generalizability of our findings. Future research should explore the experiences of women from different age groups, cultural backgrounds, and socioeconomic statuses, as well as the intersectional factors (e.g., race and sexuality) that may influence GD.

## Conclusion

This study highlights the urgent need to address gender disparities in GD research and clinical practice, with the need to reduce barriers to help-seeking among women with GD.

Women’s gaming behaviors and motivations often differ from those of men, yet diagnostic tools, research, and support systems remain largely focused on male experiences. By centering women’s voices, this research can contribute to a more inclusive and equitable understanding of GD, emphasizing the importance of women-centered approaches in diagnosis, treatment, and public health strategies. These findings support the development of women-centered diagnostic approaches, targeted awareness initiatives, and accessible support pathways to reduce barriers to help-seeking among women with GD.

Moving forward, several priorities emerge for researchers, clinicians, and policymakers, such as developing diagnostic instruments that are gender-inclusive, capturing the heterogeneous manifestations of GD; addressing stigma and the phenomenon of double sexism within both gaming culture and clinical contexts; integrating emotional regulation as a core treatment component, particularly for women whose gaming often serves as a coping mechanism; and designing gender-responsive resources and support systems, ensuring accessibility and relevance for women affected by GD.

In line with recent recommendations for person-centered and gender-sensitive care models [30], future service delivery should integrate gender-specific examples, such as the ReConnecte Programme in Switzerland [29], which illustrates how tailored interventions can address the unique needs of women with behavioral addictions.

By addressing these gaps, the field can progress toward gender-inclusive diagnostic and therapeutic frameworks, thereby ensuring that individuals of all genders have equitable access to recognition, care, and support in addressing problematic gaming behaviors.

## Data Availability

The datasets generated and analyzed during the current study are not publicly available due to the sensitive nature of the qualitative data and the need to protect participants’ confidentiality. However, it may be available from the corresponding author upon reasonable request.

## Long descriptions

### Table 1 Long description

The table consists of seven columns and eight rows including the header.

Column headers from left to right: Participant, Age, Occupation, Preferred games, Platform, Weekly gaming hours, and Self-identifies as gamer.

Row 1: Participant 1 G, age 23, Student Medicine, R P G and Visual Novels, Console, 12 to 15 hours, No.

Row 2: Participant 2 G, age 25, Student Game Art, Simulation and Strategy, P C, 15 to 20 hours, Yes.

Row 3: Participant 3 G, age 24, Student, M M O R P G and F P S, Console, 10 to 14 hours, Partially.

Row 4: Participant 4 G, age 26, Psychologist, R P G and Otome Games, P C, 18 to 22 hours, Yes.

Row 5: Participant 5 G, age 22, Student, F P S and M O B A, P C, 20 to 25 hours, Yes.

Row 6: Participant 6 G, age 21, Student, Simulation and Puzzle, Console, 8 to 12 hours, No.

Row 7: Participant 7 G, age 23, Student, R P G and Adventure, P C, 14 to 18 hours, Partially.

Navigate back to Table 1.

### Figure 1 Long description

The flowchart is organized into five dashed boundary boxes.

1. Data collection at the top includes four sequential steps: 1. Participant Recruitment, 2. Informed Consent, 3. Semi-structured Interviews, and 4. Audio Recording.

2. Data preparation follows with: 5. Transcription (TurboScribe) and 6. Data Familiarisation.

3. Analysis contains: 7. Initial Coding, 8. Theme Development, and 9. Theme Refinement.

4. Reporting at the bottom consists of: 10. Reporting and Contextualisation.

5. Review and rigour (ongoing) is positioned to the right of the main flow, containing: 11. Peer Debriefing and 12. Reflexivity and Bias Check.

Iterative feedback loops are indicated by dotted arrows. A dotted arrow labeled reflexive notes points from Reflexivity and Bias Check to Initial Coding. A dotted arrow labeled feedback points from Peer Debriefing to Theme Refinement. Additional dotted lines connect Reflexivity and Bias Check to both Theme Refinement and Reporting and Contextualisation.

Navigate back to Figure 1.

### Table 2 Long description

The table consists of three columns: Theme, Subtheme, and Dataset support across participants.

1. Theme: Identity and stigma in gaming culture.

- Subtheme: Self-identification as a gamer. Support: Yes equals 3, Partially equals 2, No equals 2.

- Subtheme: Sexism and exclusion in gaming spaces. Support: Reported across 5 interviews.

2. Theme: Gaming behaviors and motivations.

- Subtheme: Preference for at least one narrative-driven or less competitive genre such as R P G, visual novel, simulation, puzzle, adventure, or otome. Support: 5 out of 7.

- Subtheme: Gaming used for emotional coping, escape, or stress reduction. Support: Reported across several interviews.

3. Theme: Barriers to recognizing G D and accessing care.

- Subtheme: Prior familiarity with the term G D before the interview. Support: None reported (0 out of 7).

- Subtheme: Previous professional help-seeking for G D. Support: None reported (0 out of 7).

- Subtheme: Perceived lack of tailored resources for women with G D. Support: Reported across several interviews.

Navigate back to Table 2.

## References

[1] World Health Organization. (2022). International Classification of Diseases (11th Revision). https://icd.who.int

[2] Kim HS, Son G, Roh EB, Ahn WY, Kim J, Shin SH, et al. Prevalence of gaming disorder: a meta-analysis. Addict Behav. 2022;126:107183. doi:10.1016/j.addbeh.2021.107183. 34864436

[3] Hernández-Vásquez A, Vargas-Fernández R, Visconti-Lopez FJ, Comandé D, Bendezu-Quispe G. Prevalence and factors associated with gaming disorder in Latin America and the Caribbean: a systematic review. Int J Environ Res Public Health. 2022;19(16):10036. doi:10.3390/ijerph191610036. 36011671 PMC9408645

[4] Notari L, Al Kurdi C, Delgrande Jordan M, Sivanesan N Jeux de hasard et d’argent, gaming, sexualité, achats, réseaux sociaux et Internet: des conduites addictives sans substance? État des lieux sur les évidences scientifiques, la terminologie, les échelles de mesure et les prévalences. Lausanne (CH): Addiction Suisse & GREA; 2022 Jan 17. Report No 136a. ISBN 978–2–88183-279-6.

[5] Pontes, H. M., & Griffiths, M. D. (2015). *Internet Gaming Disorder Scale--9-Item Short Form (IGDS-SF9, IGDS9-SF)* [Database record]. APA PsycTests. doi:10.1037/t56703-000

[6] Lopez-Fernandez O, Williams AJ, Kuss DJ. Measuring women gaming: gamer profile, predictors, prevalence, and characteristics from psychological and gender perspectives. Front Psychol 2019;10:898. doi:10.3389/fpsyg.2019.00898. 31105622 PMC6498967

[7] King DL, Chamberlain SR, Carragher N, Billieux J, Stein D, Mueller K, et al. Screening and assessment tools for gaming disorder: a comprehensive systematic review. Clin Psychol Rev. 2020;77:101831. doi:10.1016/j.cpr.2020.101831. 32143109

[8] Carragher N, Billieux J, Bowden-Jones H, Achab S, Potenza MN, Rumpf HJ, et al. Brief overview of the WHO collaborative project on the development of new international screening and diagnostic instruments for gaming disorder and gambling disorder. Addiction. 2022;117(7):2119–21. doi:10.1111/add.15780. 34882889

[9] Dong G, Potenza MN. Considering gender differences in the study and treatment of internet gaming disorder. J Psychiatr Res. 2022;153:25–9. doi:10.1016/j.jpsychires.2022.06.057. 35793576

[10] Kuss DJ, Kristensen AM, Williams AJ, Lopez-Fernandez O. To be or not to be a female gamer: a qualitative exploration of female gamer identity. Int J Environ Res Public Health. 2022;19(3):1169. doi:10.3390/ijerph19031169. 35162194 PMC8835226

[11] Achab S Dealing with gender bias in treatment demands regarding internet-related disorders. J Behav Addict. 2022;11(1):185–188.

[12] Farhoudian A, Razaghi E, Hooshyari Z, Noroozi A, Pilevari A, Mokri A, et al. Barriers and facilitators to substance use disorder treatment: an overview of systematic reviews. Subst Abuse Res Treat. 2022;16:11782218221118462. doi:10.1177/11782218221118462. PMC9434658 36062252

[13] Park JJ, Wilkinson-Meyers L, King DL, Rodda SN. Person-centred interventions for problem gaming: a stepped care approach. BMC Public Health. 2021;21(1):872. doi:10.1186/s12889-021-10749-1. 33957877 PMC8101229

[14] Tong A, Sainsbury P, Craig J. Consolidated criteria for reporting qualitative research (COREQ): a 32-item checklist for interviews and focus groups. Int J Qual Health Care. 2007;19(6):349–57. doi:10.1093/intqhc/mzm042. 17872937

[15] Heidari S, Babor TF, De Castro P, et al. Sex and gender equity in research: rationale for the SAGER guidelines and recommended use. Res Integr Peer Rev. 2016;1(1):2. doi:10.1186/s41073-016-0007-6. 29451543 PMC5793986

[16] Braun V, Clarke V. Conceptual and design thinking for thematic analysis. Qual Psychol. 2022;9(1):3–26. doi:10.1037/qup0000196.

[17] Malterud K, Siersma VD, Guassora AD. Sample size in qualitative interview studies. Qual Health Res. 2016;26(13):1753–60. doi:10.1177/1049732315617444. 26613970

[18] TurboScribe [software]. London (UK): TurboScribe Ltd; 2024. Available from: https://turboscribe.ai

[19] Braun V, Clarke V. Reflecting on reflexive thematic analysis. Qualitative Research in Sport, Exercise & Health. 2019;11(4):589–97. doi:10.1080/2159676X.2019.1628806.

[20] Billieux J, Van der Linden M, Achab S, Khazaal Y, Paraskevopoulos L, Zullino D, et al. Why do you play world of warcraft? An in-depth exploration of self-reported motivations to play online and in-game behaviours in the virtual world of Azeroth. Comput Hum Behav. 2013;29(1):103–9. doi:10.1016/j.chb.2012.07.021.

[21] Labrador FJ, Fernández-Arias I, Martín-Ruipérez S, Bernaldo-de-Quirós M, Vallejo-Achón M, Sánchez-Iglesias I, Labrador M, Estupiñá FJ Women and videogames: what do they play? An Psicol 2022 Aug 27;38(3):508–517. doi:10.6018/analesps.504281

[22] Achab S, Nicolier M, Mauny F, Monnin J, Trojak B, Vandel P, et al. Massively multiplayer online role-playing games: comparing characteristics of addict vsnon-addict online recruited gamers in a French adult population. BMC Psychiatry. 2011;11(1):144. doi:10.1186/1471-244X-11-144. 21871089 PMC3176476

[23] Achab S, Bilke-Hentsch O, Cattin N, Messerli C. *Analyse critique sur la terminologie du rapport « Jeux de hasard et d’argent* *, gaming*, *sexualité* , *achats* , *réseaux sociaux et* *Internet :* *des conduites addictives sans substance* *?* *État des lieux sur les évidences scientifiques* , *la terminologie* , *les échelles de mesure et les prévalences* *»* GREA Fachverbansucht, 2022. https://www.addictionsuisse.ch/wp-content/uploads/2023/11/Analyse-critique-terminologie-rapport-addictions-comportementes-.pdf

[24] Lopez-Fernandez O, Williams AJ, Griffiths MD, Kuss DJ. Female gaming, gaming addiction, and the role of women within gaming culture: a narrative literature review. Front Psych. 2019;10:454. doi:10.3389/fpsyt.2019.00454. PMC6635696 31354536

[25] Castro CM, Neto DD. From healthy play to gaming disorder: psychological profiles from emotional regulation and motivational factors. J Behav Addict. 2025;14(1):276–88. Published 2025 Jan 17. doi:10.1556/2006.2024.00077. 39819889 PMC11974420

[26] Castro CM, Neto DD. Profiling gamers: the role of mental health, attachment and social factors in gaming behaviors. Addict Behav. 2025;169:108390. doi:10.1016/j.addbeh.2025.108390. 40414138

[27] Achab S, Rothen S, Giustiniani J, Nicolier M, Franc E, Zullino D, et al. Predictors of gaming disorder or protective from it, in a French sample: a symptomatic approach to self-regulation and pursued rewards, providing insights for clinical practice. Int J Environ Res Public Health. 2022;19(15):9476. doi:10.3390/ijerph19159476. 35954855 PMC9367741

[28] Kuss DJ, Kristensen AM, Williams AJ, Lopez-Fernandez O. To be or not to be a female gamer: a qualitative exploration of female gamer identity. Int J Environ Res Public Health. 2022;19(3):1169. doi:10.3390/ijerph19031169. 35162194 PMC8835226

[29] Achab S. Réinventer le soin des addictions comportementales: la médecine centrée Sur la personne. Rev Med Suisse. 2025;21(917):996. doi:10.53738/REVMED.2025.21.917.47093. 40336241

[30] Achab S. Gender-specific personalized care delivery for problematic use of internet in Switzerland. In: Prever F, Blycker G, Brandt L, editors. Behavioural addiction in women: an international women perspective on treatment and research. 1st ed. Routledge; 2023, p. 138–44. doi:10.4324/9781003203476.

